# Right Gaze Palsy and Hoarseness: A Rare Presentation of Mediastinal Tuberculosis with an Isolated Prepontine Cistern Tuberculoma

**DOI:** 10.1155/2015/718289

**Published:** 2015-11-29

**Authors:** Chidozie Charles Agu, Olufemi Aina, Md Basunia, Bikash Bhattarai, Vikram Oke, Marie Frances Schmidt, Joseph Quist, Danilo Enriquez, Vijay Gayam

**Affiliations:** Interfaith Medical Center, Brooklyn, NY, USA

## Abstract

We describe a previously healthy young man who presented with headaches, diplopia with right lateral gaze palsy, dysphagia, and hoarseness over a 2-month period. Magnetic resonance imaging of the brain revealed a small enhancing mass at the prepontine cistern and chest CT showed a left mediastinal mass. Mediastinoscopy and lymph node biopsy were performed. DNA probe and culture of the biopsy specimen were confirmed to be* Mycobacterium tuberculosis* complex. Resolution of neurologic symptoms was noted after 6 weeks, in addition to regression of brain stem and mediastinal lesions after 12 weeks of antituberculous therapy.

## 1. Introduction

Mediastinal lymphadenopathy as a manifestation of tuberculosis (TB) is a relatively common entity in developing countries [1]. However, involvement of the recurrent laryngeal nerve without associated chronic pleuropulmonary lung disease is rare and only few cases have been reported in medical literature [2]. While CNS tuberculomas are a relatively common manifestation of CNS tuberculosis in endemic regions, isolated prepontine cistern tuberculomas are very rare and may be associated with cranial nerve palsies [3]. We herein describe a case of a young immunocompetent male presenting with left vocal cord palsy and right gaze palsy resulting from mediastinal lymphadenitis associated with an isolated prepontine cistern tuberculoma.

## 2. Case Report

A 32-year-old male African immigrant presented with severe early morning headaches, diplopia on lateral gaze, hoarseness, and mild dysphagia (to both solids and liquids).

His symptoms had progressively worsened over the course of 2 months. He also complained of one week of drenching night sweats but no fever, fatigue, or weight loss. His past medical history was remarkable for recent migration from Nigeria about five years ago and a positive tuberculin test. Physical examination was remarkable for limited abduction of the right eye during horizontal gaze (right abducens palsy), but pupillary reflexes were normal and there was no papilledema on fundoscopy. There were no meningeal signs and no other neurological deficits on exam. Examination of the ears, nose, and throat was unremarkable and there were no palpable lymph nodes.

**Figure 1:**
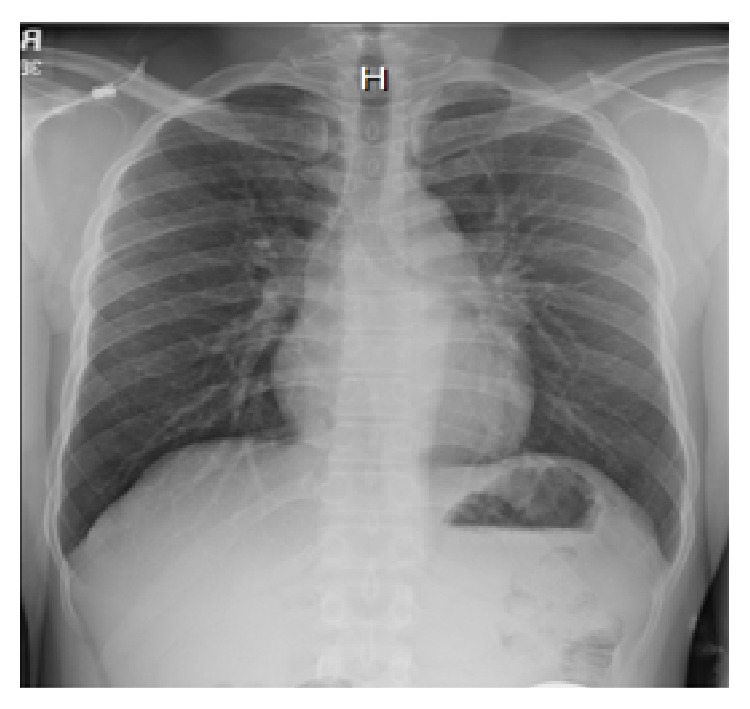
Chest X-ray (PA view) with left hilar fullness but no pulmonary lesions.

**Figure 2:**
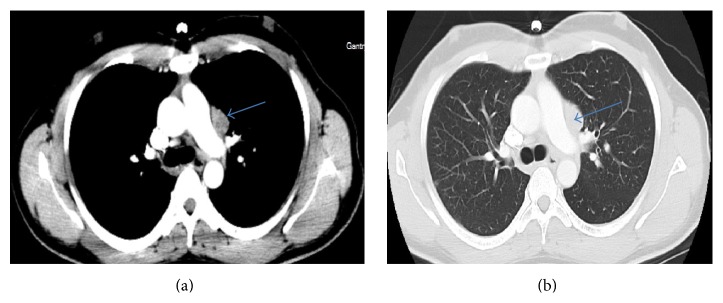
Axial CT scans of the chest ((a) mediastinal window; (b) lung window) showing large mediastinal lymph node with normal lung parenchyma before anti-TB therapy.

Complete blood count, blood chemistries, liver function tests, coagulation profile, thyroid function tests, serum vitamin B12, folate, and angiotensin-converting enzyme (ACE) levels were within normal limits. Erythrocyte sedimentation rate (ESR) and C-reactive protein were elevated at 36 mm/h (0–15 mm/h) and 22.4 mg/dL (0–4.9 mg/dL), respectively. QuantiFERON test was positive, while syphilis, Lyme, and HIV serologies were negative. Autoimmune workup including antinuclear antibody and anti-neutrophil cytoplasmic antibody (ANCA) panel was negative. Chest X-ray showed some fullness in the left hilar region but no pulmonary parenchymal lesions (Figure 1). Noncontrast CT brain was unremarkable. Contrast chest CT was notable for a left AP window mass measuring 3 × 1.8 × 2.7 cm (Figures 2(a) and 2(b)). Abdominal/pelvic CT was unremarkable. MRI of brain with gadolinium revealed an enhancing mass of approximately 7 × 17 × 9 mm effacing the prepontine cistern and contacting the ventral aspect of the right side of the pons (Figure 4). There was no surrounding edema or midline shift. A spinal tap was done with an opening pressure of 34 cm H20, and clear, colorless cerebrospinal fluid (CSF) was obtained. CSF analysis was as follows: WBC 0/*μ*L (0–5), RBC 0/*μ*L (0–5), glucose 67 mg/dL (40–70), total protein 49 mg/dL (15–45), VDRL negative, Lyme titers negative, HSV I/II DNA PCR negative, toxoplasma IgM antibody negative, cryptococcus antigen negative, and no atypical/malignant cells. CSF gram stain and cultures as well as acid-fast stain and DNA probe for* Mycobacterium tuberculosis* were negative.

Flexible bronchoscopy revealed a fixed left vocal cord. No endobronchial lesions were visualized. Bronchoalveolar lavage (BAL) was negative for acid-fast bacilli (AFB) and fungal stains, while cytology was negative for malignant cells. The mediastinal node station was inaccessible by endobronchial ultrasound (EBUS). Mediastinoscopy with lymph node biopsy was done revealing granulomatous disease with focal necrosis. AFB and fungal stains of the lymph node were negative.

**Figure 3:**
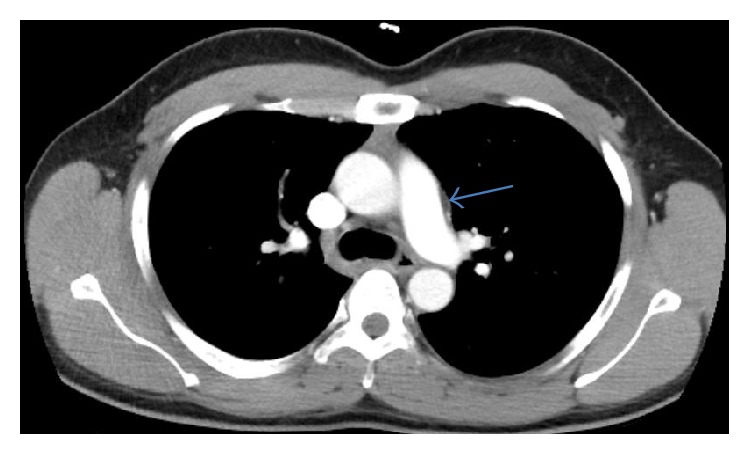
Axial CT scan of the chest (mediastinal window) with regression of mediastinal mass after several months of anti-TB therapy.

**Figure 4:**
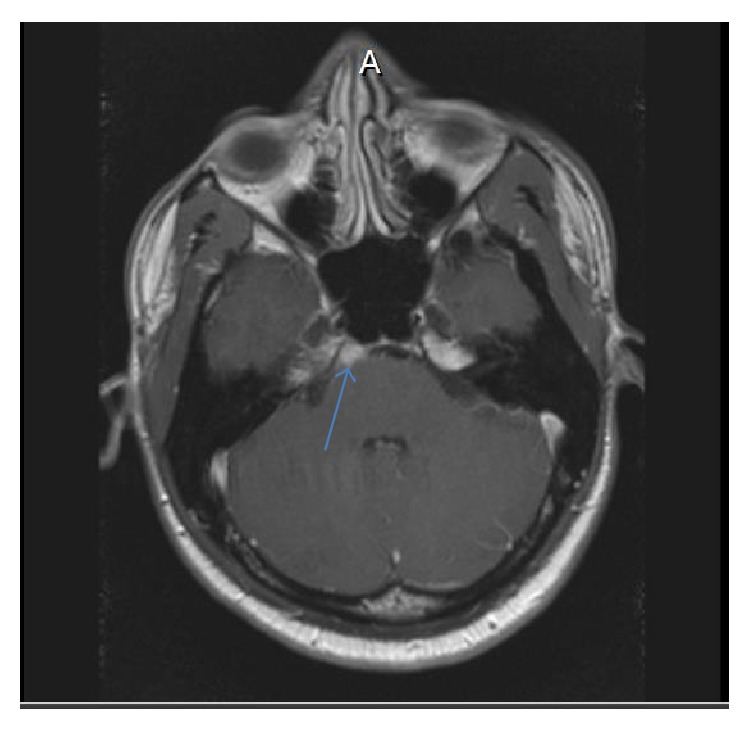
MRI of brain T1 axial + gadolinium showing small tuberculoma in the right prepontine cistern before anti-TB treatment.

**Figure 5:**
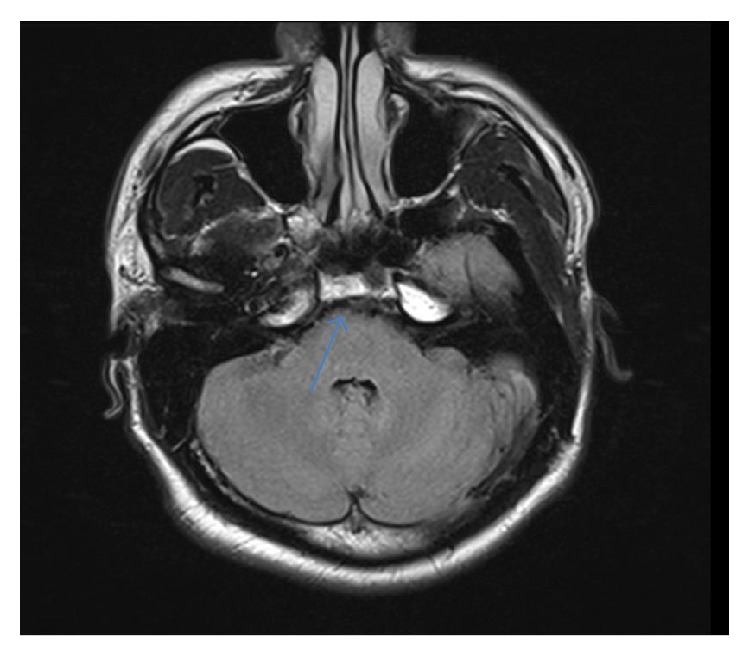
MRI of brain T1 axial + gadolinium with resolution of previously visualized enhancing lesion, after anti-TB treatment.

Empirically, antituberculous treatment with isoniazid, rifampin, pyrazinamide, and ethambutol was instituted due to high suspicion of TB. DNA probe and culture later returned positive for pansensitive* Mycobacterium tuberculosis* complex. Stereotactic brain biopsy was not performed as the brain stem lesion was presumed to be a tuberculoma. A short course of corticosteroids (one month) was administered. After 6 weeks of treatment, the headaches, diplopia, dysphagia, and hoarseness had resolved and repeat brain MRI and chest CT at 12 weeks showed interval regression of mediastinal and brain lesions (Figures 3 and 5, resp.). The patient remained asymptomatic and completed 12 months of antituberculous therapy.

## 3. Discussion

Vocal cord paralysis is due to the involvement of recurrent laryngeal nerve by benign.

Inflammatory mediastinal lymphadenopathy is quite rare [4]. In industrialized countries, this is mostly related to bronchogenic carcinoma or lymphoma [2, 4, 5]. Left recurrent laryngeal nerve (RLN) injury has been associated with chronic fibrosing pulmonary TB, which could be as a result of accompanying enlarged tuberculous mediastinal lymph nodes, recurrent laryngeal nerve entrapment in the dense fibrous pleural thickening, and fibrosing mediastinitis or as a result of the nerve being stretched owing to retraction of the left upper lobe bronchus towards the apex [2, 4]. Left RLN involvement solely due to focal mediastinal tuberculous lymphadenopathy without accompanying pleuropulmonary disease or diffuse mediastinal involvement is very rare and therefore malignancy must first be excluded [4, 6]. Cytological and histological examinations show classic caseating necrosis only in about 50% of tuberculous lymphadenitis [1] and therefore the absence of these findings does not exclude tuberculosis. Other granulomatous diseases including sarcoidosis and fungal infections should also be considered. In our patient, we believe that the location of enlarged tubercular nodes in the confined space of the aortopulmonary window and arch of the aorta predisposed to compression and dysfunction of the left recurrent laryngeal nerve leading to left vocal cord paralysis, hoarseness, and dysphagia.

Tuberculomas typically occur as a result of hematogenous spread to the central nervous system (CNS) from a primary tuberculous focus and manifest clinically as cranial nerve palsies and other focal neurological signs of brain stem involvement depending on their location [7–10]. They are typically seen in immunocompromised individuals, who reside in highly endemic areas [11]. Tuberculomas located solely in the prepontine cistern are very rare and only one case has been reported so far. Yanardag et al. reported the case of an isolated prepontine tuberculoma in a young male presenting with right ptosis and diplopia [3].

Diagnosis of CNS tuberculomas is based on clinical findings and brain imaging. Stereotactic brain biopsy with histopathological examination may be required in the face of diagnostic uncertainty [12]. Differentials are broad including neoplastic (both primary and metastatic), parasitic, and fungal lesions especially in immunocompromised individuals. CSF studies are usually nonspecific in the absence of concomitant meningitis. Elevated CSF protein is the most consistent finding, while CSF AFB stains and cultures are usually negative [11, 12]. PCR for MTB DNA was positive in one case series [12]. In the absence of stereotactic brain biopsy, response to empirical anti-TB therapy makes the diagnosis almost certain [11].

A six- to nine-month regimen (two months of isoniazid, rifampin, pyrazinamide, and ethambutol, followed by four to seven months of isoniazid and rifampin) is recommended as initial therapy for all forms of extrapulmonary tuberculosis unless the organisms are known or strongly suspected to be resistant to the first-line drugs [13]. For CNS TB, the Center for Disease Control and Prevention (CDC) recommends 12 months of treatment (two months of isoniazid, rifampin, pyrazinamide, and ethambutol, followed by ten months of isoniazid and rifampin) when the mycobacterial strain is sensitive to all drugs [13, 14]_._ It has also been suggested that treatment duration be tailored to radiological response [14, 15]. After 12 months of treatment, about two-thirds of the patients may still have contrast enhancing brain lesions, though it is not clear if this represents an active lesion or just residual inflammation. Continuing treatment would be prudent in this situation [14, 15]. Systemic corticosteroids as adjuvant therapy are indicated when there is perilesional edema, elevated intracranial pressure, or paradoxical worsening due to enlargement of lesions during treatment [12, 14]. Total resolution of the tuberculoma is observed when MRI images demonstrate no enhancing lesions or only an area of calcification [14, 15]. In our case, the diplopia and right lateral gaze palsy were likely as a result of a prepontine cistern tuberculoma compressing the right pontine base at the origin of the right abducens nerve (VI). This case is unique because there have been no reported cases of vocal cord and abducens nerve paralysis as a result of concomitant mediastinal tuberculous lymphadenopathy and an isolated prepontine cistern tuberculoma in an immunocompetent individual. It also highlights the importance of vigilance in the diagnosis of atypical presentations of tuberculosis in immunocompetent individuals migrating from highly endemic regions.
